# When images don’t explain reality: A case of an intravascular extension of an endometrial tumor

**DOI:** 10.21542/gcsp.2023.11

**Published:** 2023-05-11

**Authors:** Kyle Marrache, Hussam Al Hennawi, Shayan Qadir, Angad Bedi, Kevin Ye Hou

**Affiliations:** 1 Jefferson Abington Hospital, Abington, Pennsylvania, USA; 2 Asplundh Cancer Pavilion – Sidney Kimmel Cancer Center, Willow Grove, Pennsylvania, USA

## Abstract

Endometrial stromal sarcoma is a rare mesenchymal tumor with the potential for intravascular metastases. We present the case of a 52-year-old female patient with a history of previously resected endometrial sarcoma who was found to have an inferior vena cava thrombus with intracardiac extension. Pathological assessment following resection revealed intravascular extension of an underlying endometrial stromal sarcoma. This case demonstrates the aggressive nature and intracardiac metastatic potential of endometrial sarcomas.

## Background

Endometrial stromal sarcoma (ESS) accounts for approximately 0.2% of all malignant uterine tumors. Considered a rare malignant mesenchymal tumor, ESS accounts for 10–15% of all uterine sarcomas, with an annual incidence of 1–2 per million women^1, 2^. Although occasionally observed in other malignancies, such as renal cell carcinoma, malignant extension of ESS into the IVC is rare. In this report, we describe a complex case of a large-burden IVC tumor thrombus stemming from an underlying ESS with extension from the iliac veins through the entire IVC, right atrium, and right ventricle before ending in the proximal pulmonary artery. This case also brings to light educational imaging and interventional approaches for rare, high-risk cases.

## Case presentation

**Figure 1.**
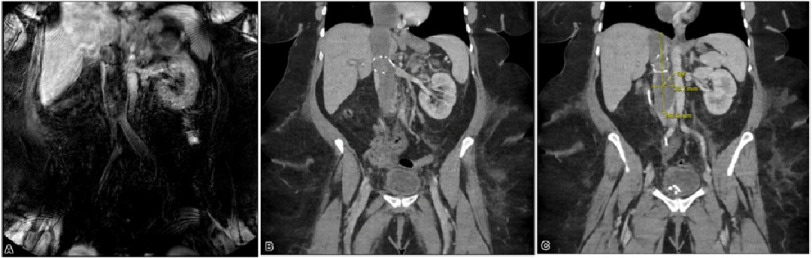
MRI venography showing occlusive bland inferior vena cava thrombus distending the IVC and extending to the right atrium and ventricle (A). CT scan depicting the dilated inferior vena cava, with IVC filter at the superior aspect. Findings are suspicious for a non-obstructing thrombus at this level (B,C).

A 52-year-old female patient was initially admitted after pre-operative blood tests before routine ureteral stent replacement showed platelet count of 10 × 10^3^/mL . She had a significant medical history of endometrial stromal sarcoma managed with partial hysterectomy, followed by adjuvant hormonal therapy with letrozole and progesterone. She also had a history of a massive pulmonary embolism after hysterectomy requiring thrombectomy with IVC filter placement followed by long-term anticoagulation, chronic right ureteral stent replacement every 6 months, surgically repaired aorta-to-right atrial fistula, and ITP. Before admission, the patient complained of worsening fatigue and shortness of breath for several months. Initial physical examination revealed +3 pitting edema of the lower extremities. Laboratory investigations showed acute kidney injury with subsequent ultrasound showing no hydronephrosis or ureteral obstruction. An MR venogram was performed to rule out an IVC thrombus obstructing the left renal vein, given her previous IVC thrombus. MRV demonstrated a bland IVC thrombus causing distension of the IVC that extended to the right atrium and ventricle (Figure 1). CT of the abdomen/pelvis confirmed these findings. Echocardiography revealed right atrial dilation with a 5.5 cm spherical well-circumscribed thrombus in the right atrium protruding into the right ventricle.

The patient was transitioned from low-dose apixaban to full-dose rivaroxaban to reduce further clot propagation, as surgical intervention was deemed high-risk, given her previous cardiothoracic surgical history and concomitant thrombocytopenia. Patient platelet count improved to 103 × 103/mL, while inpatient received IVIG and platelet transfusions. She was discharged on anticoagulation therapy and opted to seek a second opinion regarding possible clot removal.

**Figure 2.**
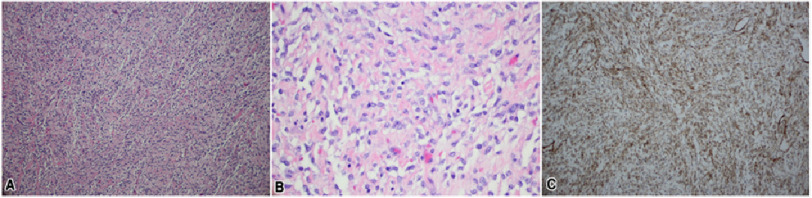
Microscopic evaluation showing a uniform population of small cells with eosinophilic cytoplasm (A). The cells are epithelioid with clear vacuolated cytoplasm and uniform round to slightly oval nuclei. Cells are clustered around capillaries. Between clusters there is abundant lacy pink proteinaceous material separating the cells. Mitoses average 6 per 10 high-power fields (B). CD10 immunostaining depicting reactivity (C).

Six days after discharge, the patient returned to the emergency room with progressive shortness of breath, which was thought to be the result of a worsening thrombus. Tissue plasminogen activator (TPA) could not be administered because of a repeat platelet count of 15 × 10^3^/mL. The patient underwent urgent surgical intervention with sternotomy under cardiopulmonary bypass. A large right atrial mass connected to the IVC mass as well as contiguous extension into the right ventricle and pulmonary artery were identified and resected at the level of the IVC entrance. The nearby IVC filter was also removed. Pathologic evaluation showed low-grade ESS with immunohistochemical staining positive for ER, PR, WT-1, CD10, and desmin, but negative for smooth muscle actin and CD31. (Figure 2).

**Figure 3.**
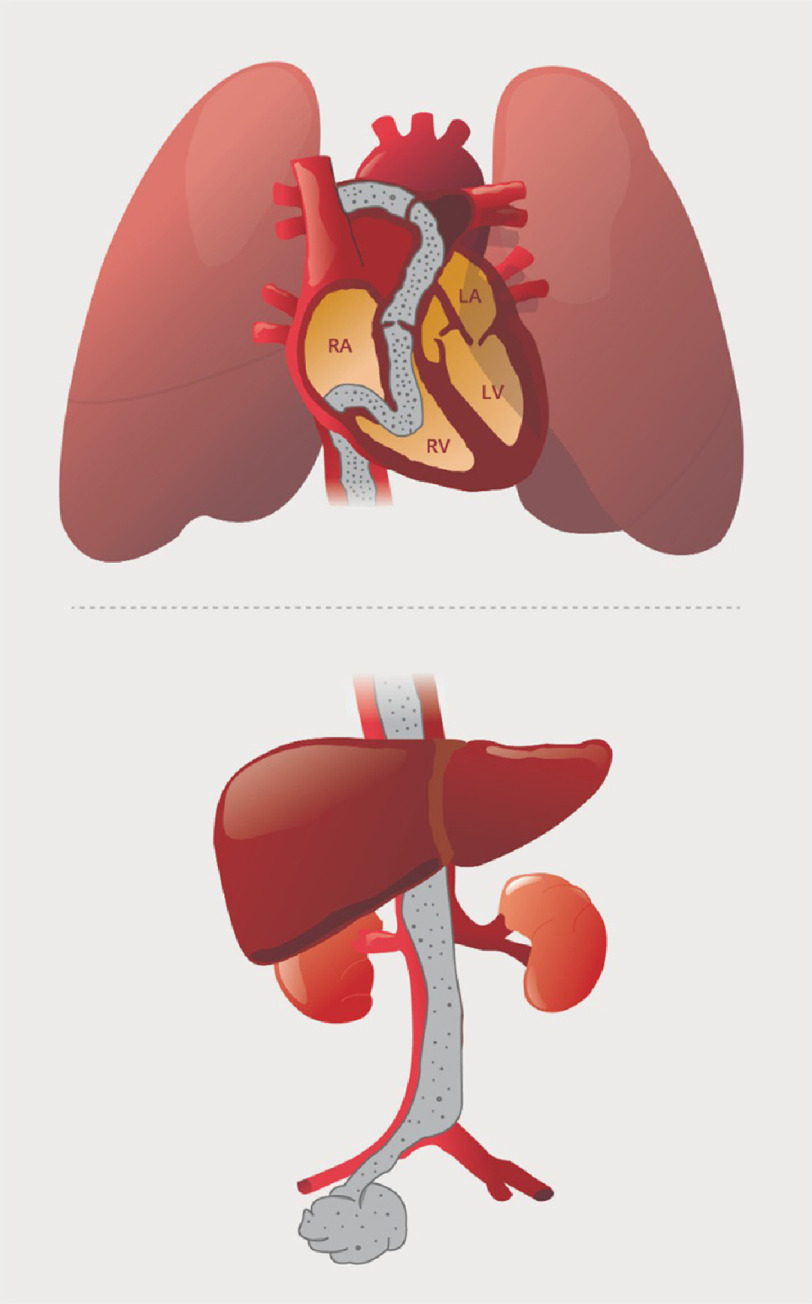
Diagram illustrating the spread of IVC tumor thrombosis.

Postoperatively, the patient developed acute shock requiring multiple vasopressors, as well as worsening renal function requiring hemodialysis. On postoperative day 1, the patient developed a significant hemothorax that required surgical chest tube placement. On postoperative day 2, the patient developed unstable SVT that deteriorated into ventricular tachycardia. Despite ACLS support with CPR and defibrillation, the patient’s ventricular arrhythmia could not be corrected, and the died.

## Discussion

In the early stages, the 5-year survival rate for ESS has been reported to range from 54% to nearly 100% but falls to 11% in those with advanced disease^1^. Late recurrences have been seen in 30–50% of cases^3^. ESS has been known to metastasize through both lymphatic and hematogenous spread (Figure 3)^4^. A potential symptom after vascular invasion is new-onset peripheral edema due to increased intravascular hydrostatic pressure secondary to blockage of the IVC, which was observed in our case (Figure 1)^5–8^. A literature review identified 19 patients with advanced low-grade ESS complicated by invasion of the great vessels and IVC tumor thrombus^9^. Among these 19 patients, four had extension to at least the right atrium, with two showing extension into the pulmonary artery^9^. The present case is another example of this rare presentation.

In terms of management, surgical resection of the tumor thrombus is the preferred approach. However, the rarity of this condition has resulted in a paucity of published studies^10^. One case study examined the management of inoperable cases with long-term anticoagulation to prevent further thrombus formation in the context of cancer metastasis and hypercoagulability^11^. The use of the AngioVac System, a mechanical aspiration device that has FDA approval for removing unwanted intravascular material during extracorporeal bypass, has been studied as an alternative to surgical resection in the setting of advanced renal cell carcinoma involving the IVC. A case study reported the unsuccessful removal of an ESS-related IVC tumor thrombus using the AngioVac System, thought to be the result of significant lumen adhesion by the tumor^12^.

## Conclusion

Endometrial stromal sarcoma is a rare condition with the potential for significant vascular invasion. Patients with metastasis to the IVC commonly present with peripheral edema and can have extensive IVC tumor thrombosis, potentially extending into the heart chambers and pulmonary artery. Surgical thrombectomy, with or without extracorporeal bypass, is the mainstay of treatment, but carries a significant surgical risk. Inoperable cases are typically managed with long-term therapeutic anticoagulation to prevent further propagation of tumor thrombus. The use of the AngioVac System and other aspiration cannulas represents a novel option in high-risk surgical cases and warrants further evaluation.
